# Dose-dependent exercise promotes the transition from atrial protection to arrhythmogenic vulnerability: the intermediary role of calcium handling instability in substrate remodeling

**DOI:** 10.3389/fphys.2026.1878610

**Published:** 2026-08-31

**Authors:** Shuwei Suo, Xiaolin Zhou, Yan Luo, Zhen Zhang, Junli Pan, Huaxin Sun, Hanxiong Liu

**Affiliations:** Department of Cardiology, The Third People’s Hospital of Chengdu (Affiliated Hospital of Southwest Jiaotong University), College of medicine, Southwest Jiaotong University, Chengdu, Sichuan, China

**Keywords:** atrial fibrillation, calcium handling, endurance exercise, heart rate variability, optical mapping

## Abstract

**Background:**

Endurance athletes carry an elevated risk of atrial fibrillation (AF), yet the exercise dose threshold at which physiological cardiac adaptation gives way to maladaptive remodeling, and the potential involvement of calcium (Ca^2+^) handling in this transition, remain poorly delineated.

**Methods and results:**

C57BL/6 mice were stratified into sedentary control, low-, moderate-, and high-dose forced treadmill groups for 10 weeks. A modified eight-parameter behavioral panel confirmed progressively compromised exercise tolerance in the high-dose group. Surface ECG revealed significant P-wave morphological alterations and PR interval shortening, while HRV analysis demonstrated sympathovagal co-activation confined to the high-dose group. Echocardiography identified adverse atrial geometric remodeling, and histopathology demonstrated extensive fibrosis with inflammatory infiltration. Programmed electrical stimulation uncovered a striking dose-response gradient: AF inducibility reached 62.5% in the high-dose group versus 0% in the moderate-dose group. RNA sequencing identified 758 - 2,237 DEGs across the three comparisons, of which 13.6 - 17.2% were annotated to Ca^2+^-regulatory processes. KEGG and cluster analyses revealed enrichment of Ca^2+^ signaling genes in both up- and down-regulated gene modules, while protein-protein interaction (PPI) network analysis suggested that inter-group transcriptional differences were largely related to calcium handling, cardiac contraction, myocardial remodeling, adrenergic signaling, autonomic regulation, and ECM remodeling. Optical mapping delineated dose-dependent remodeling of atrial Ca^2+^ transient dynamics, accompanied by concordant action potential duration alterations.

**Conclusion:**

High-dose exercise is associated with progressive maladaptive atrial remodeling encompassing structural, electrical, and Ca^2+^ handling alterations. Impaired Ca^2+^ release-reuptake balance and transcriptional alterations in Ca^2+^ signaling pathways are potentially linked to a pro-arrhythmic substrate, suggesting that Ca^2+^ handling instability may serve as a plausible intermediary between high-dose exercise and increased arrhythmogenic vulnerability.

## 1 Introduction

Atrial fibrillation (AF) is the most prevalent type of sustained cardiac arrhythmia, affecting more than 50 million individuals worldwide and substantially increasing the risk of stroke, heart failure, and mortality (Andrade et al., 2025; Meyer et al., 2025). The increased risk of AF in athletes is a classic topic in the field of AF research. The incidence of AF among high-dose endurance athletes, particularly long-distance runners, cyclists, cross-country skiers, and swimmers, is 2 to 10-fold greater than that among nonathletic individuals (Palermi et al., 2025; Myrstad, 2026). Notably, a recent genomic analysis in elite rowers revealed that the exercise-induced arrhythmic substrate operates through mechanisms substantially independent of inherited polygenic risk (Flannery et al., 2025).

Endurance exercise is also a modifiable risk factor for the broader population of physically active individuals, not solely for competitive athletes. Physical activity is recognized as a cornerstone lifestyle intervention for cardiovascular disease prevention. In the general adult population, epidemiological evidence consistently demonstrates that guideline-concordant moderate-dose exercise confers robust cardioprotection (Lee et al., 2022; Lloyd-Jones et al., 2022; Van Gelder et al., 2024). However, unaccustomed high-dose exertion, particularly in deconditioned nonathletes, may paradoxically precipitate the very cardiovascular events it is intended to prevent (Franklin et al., 2020).

The relationship between exercise level and AF susceptibility is best described by a “U-shaped” or “J-shaped” curve, in which both physical inactivity and excessive endurance exercise confer increased arrhythmic risk, whereas moderate physical activity has a protective effect (Meza-Ramos et al., 2023; Xiao et al., 2025). At the left end of this curve, epidemiological evidence indicates that moderate levels of physical activity reduce the risk of atrial fibrillation (Liu et al., 2024; Chen et al., 2025). At the right end, the protective effect is reversed: greater cumulative exercise levels confer a higher risk of AF (Svedberg et al., 2019; Lenting et al., 2024). These converging clinical data indicate that the exercise-AF relationship is continuous and dose dependent, with a definable transition zone beyond which cardiovascular benefit gives way to arrhythmogenic risk.

The biological mechanisms underlying exercise-induced AF have been investigated through a series of animal studies that have collectively focused on structural remodeling and inflammation. whereas Aschar-Sobbi et al. and Lakin et al. reported that tumor necrosis factor-alpha (TNFα) is the principal driver of exercise-induced adverse atrial remodeling (Aschar-Sobbi et al., 2015; Lakin et al., 2023). Gorman et al. provided the first direct evidence of dose dependent, atrial-specific vulnerability: graded daily swim durations progressively increased atrial hypertrophy and fibrosis in mice, accompanied by macrophage accumulation (Gorman et al., 2024).

Despite these advances, several critical gaps persist. First, the diversity of animal models (Guasch et al., 2013), spanning different species (rats, mice, and pigs), exercise modalities (treadmill, swimming, and wheel running), training durations (4–16 weeks), and dose prescriptions, coupled with the absence of standardized criteria for defining exercise dose, has limited cross-study comparability and precluded the identification of precise dose-response thresholds (Schuttler et al., 2020). Second, existing studies have predominantly employed dichotomous comparisons (exercise vs. sedentary) or varied exercise durations at a fixed dose (Guasch et al., 2013; Gorman et al., 2024) rather than continuously examining graded exercise intensities at matched durations.

Cardiac remodeling is widely known to perturb intracellular calcium (Ca^2+^ homeostasis, a central pathogenic mechanism in arrhythmogenesis (Mattiazzi et al., 2025; Pronto et al., 2025). High-dose exercise results in sustained increases in myocardial mechanical work and oxygen consumption, placing exceptional demands on atrial Ca^2+^ cycling and energy homeostasis (Keidar et al., 2023). When exercise-induced structural remodeling superimposes on these acute hemodynamic stresses, coordinated mechanoelectrical and mechanoenergetic coupling may become progressively compromised, creating a substrate in which Ca^2+^ handling shifts from physiological adaptation to arrhythmogenic dysfunction (Soattin et al., 2026). Aschar-Sobbi et al. reported increased atrial-specific CaMKII-dependent RyR2-S2814 phosphorylation in exercised mice (Aschar-Sobbi et al., 2015), suggesting a direct link between exercise-induced remodeling and impaired Ca^2+^ handling. However, whether exercise dose produces dose-dependent alterations in the atrial calcium transcriptome and tissue-level calcium dynamics has not been comprehensively investigated.

Building upon this evidence, we propose three interrelated hypotheses. Thus, while high-dose endurance training may increase cardiopulmonary reserve through adaptive ventricular and vascular remodeling, it simultaneously induces maladaptive remodeling of atrial electrophysiological properties and Ca^2+^ handling dynamics, creating a dissociation between systemic cardiovascular fitness and AF vulnerability.

## 2 Methods

### 2.1 Mouse models and husbandry

All animal procedures were approved by the Animal Care and Use Committee of Southwest Jiaotong University (Approval No. SWJTU-2403-NSFC (085)). Because female mice are subject to the influence of cyclical estrogen fluctuations, we selected male mice, whose physiological state is more stable, for the present study. Male C57BL/6 mice (approximately 8 weeks old) were purchased from Beijing HFK Bioscience Co., Ltd. During the modeling period, mice were housed in groups according to exercise dose and maintained in a temperature- and humidity-controlled environment with natural soft fiber bedding under a 12-hour light/dark cycle. All mice had ad libitum access to standard laboratory rodent chow and water. At the conclusion of the experimental protocol, mice were euthanized by inhalation of 5% isoflurane in 100% oxygen delivered at a flow rate of 1 L/min until complete cessation of respiration, followed by cervical dislocation as a secondary method to confirm death. All procedures were performed in accordance with the AVMA Guidelines for the Euthanasia of Animals (2020 Edition), and this euthanasia protocol was uniformly applied across all experimental groups. Mice were randomly assigned to groups using computer-generated random numbers.

### 2.2 Maximum endurance speed test

Mice underwent three maximal endurance running-speed tests at 24-h intervals, with the final test completed at least 72 h before the start of the 10-week exercise training protocol. The exercise load testing protocol was designed to determine maximal running speed and was conducted as follows. Prior to formal testing, untrained mice underwent an acclimation period consisting of two 10-min sessions at 5 m/min (0% incline) on an enclosed eight-lane treadmill (SA101, SANS Biotechnology Co., Ltd.). Following acclimation, mice were placed on an enclosed single-lane treadmill for maximal exercise testing. After a 10-minute rest period, mice began running at an initial speed of 5 m/min (0% incline) for 5 minutes. Subsequently, treadmill speed was incrementally increased by 1 m/min every minute until exhaustion. Exhaustion was defined as meeting either of the following criteria: (1) remaining on the electrical stimulus grid at the rear of the treadmill for ≥5 consecutive seconds, or (2) receiving the maximum threshold of 150 electrical stimuli (Hughey et al., 2024).

### 2.3 Exercise training protocol

**Figure 1:**
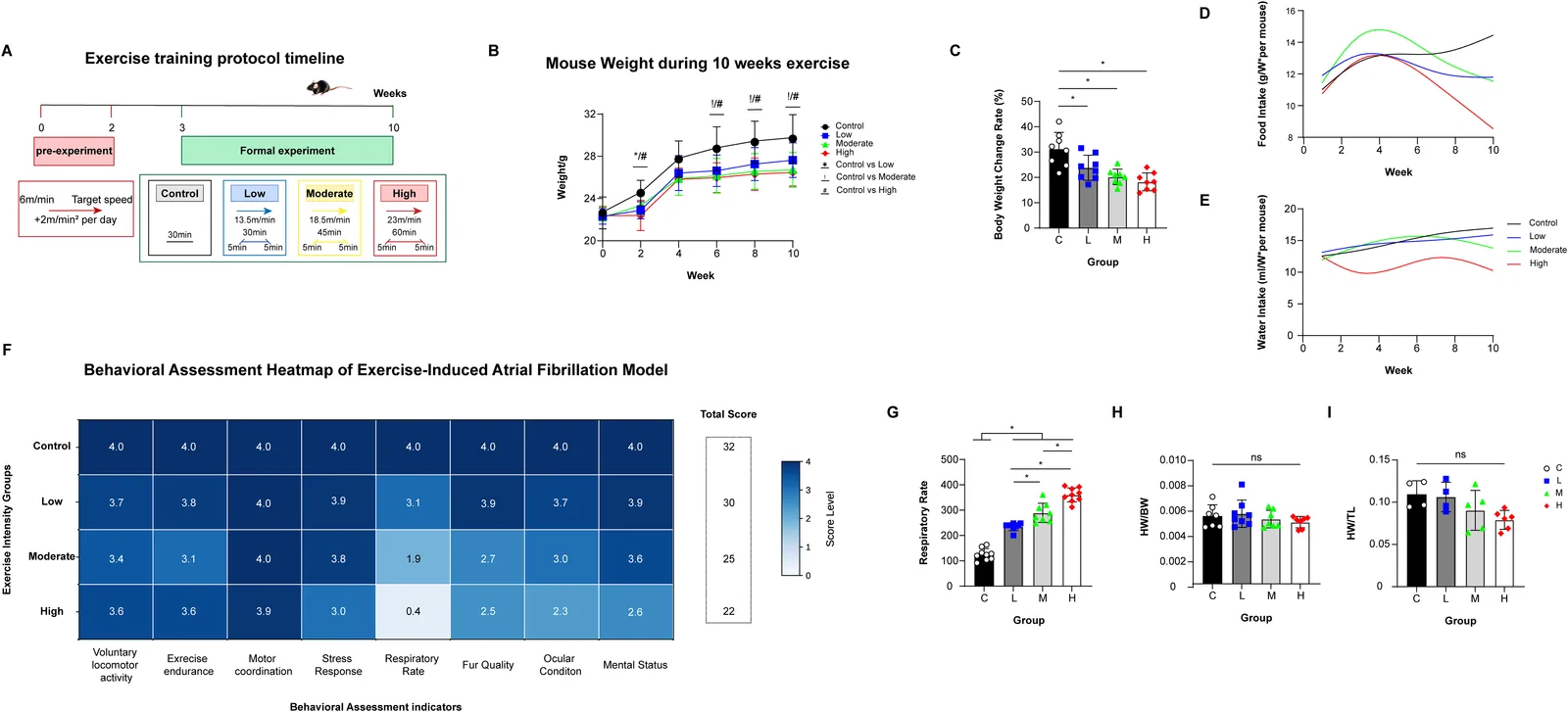
Dose-dependent effects of chronic treadmill exercise on body weight, performance, and behavioral outcomes in mice. **(A)** Timeline of the exercise protocol. **(B)** Changes in body weight over the 10-week exercise period. **(C)** Percent change in body weight between the endpoints and baseline. **(D)** Changes in food intake over the 10-week period. **(E)** Changes in water intake over the 10-week period. **(F)** Heatmap of behavioral scale scores. **(G)** Respiratory rate at the end of the exercise protocol. **(H)** Heart weight-to-body weight ratio at the endpoint. **(I)** Heart weight to tibia length ratio at the endpoint. C, Control group; L, low-dose group; M, moderate-dose group; H, high-dose group; W, week; HW, heart weight; BW, body weight; TL, tibia length. The data are presented as the mean ± SD; *P < 0.05; one-way ANOVA with Tukey’s multiple comparisons test was used. The data points represent the means at each time point, and a fitting curve was used to model the trend (D&E).

Mice underwent a two-week adaptive training period to acclimate to treadmill exercise. Training commenced at an initial speed of 6 m/min, with incremental increases of 2 m/min daily until each mouse reached its individually determined target speed. This gradual progression allowed physiological adaptation and minimized stress responses. Following the adaptive period, mice were randomly assigned to one of four experimental groups and subjected to an eight-week structured training protocol. Mice in the rest group were placed on a stationary treadmill for 30 minutes daily without running, thereby controlling for environmental exposure, whereas those in the low-, moderate-, and high-dose exercise groups underwent progressively more demanding regimens of continuous treadmill running. Specifically, the low-dose group ran for 30 minutes daily at 13.5 m/min, the moderate-dose group for 45 minutes daily at 18.5 m/min, and the high-dose group for 60 minutes daily at 23 m/min (Guo et al., 2020). Animals were exercised five days per week with two intervening rest days to allow for adequate recovery. All exercise bouts were bracketed by standardized 5-minute warm-up and cool-down periods at reduced treadmill speeds to minimize injury risk and promote physiological adaptation (**Figure 1A**).

### 2.4 Exercise assessment and monitoring

Body weight was recorded daily at consistent time points. Mice were group-housed by treatment conditions, with food and water consumption monitored every day. Exercise sessions were video-recorded, and daily observational assessments included voluntary locomotor activity, endurance, motor coordination, and stress responses (Sun et al., 2023). Post-exercise evaluations included respiratory rate (measured by visual assessment of thoracic movements for ≥1 minute), fur quality, ocular condition, and mental status. Upon completion of the 10-week intervention, heart weight was measured using an analytical balance, and tibia length was determined with digital calipers to allow heart weight normalization. Detailed behavioral assessment protocols are described in the **Supplementary Material** (**Supplementary Table 1**).

### 2.5 Surface electrocardiographic recording

Surface electrocardiogram (ECG) data were acquired at the end of each weekly exercise session (every Friday). Mice were lightly anesthetized by inhalation of 1% isoflurane in 100% oxygen at a flow rate of 1 L/min to maintain a relatively conscious state, and core body temperature was maintained between 36.5 °C and 37.5 °C using a thermostatically controlled heating pad. Following a 15-minute stabilization period after the cessation of exercise, limb-lead electrodes were attached to all four extremities to obtain surface ECG recordings in the supine position. Signals were acquired using a BioAmp amplifier (AD Instruments, Dunedin, New Zealand), digitized, and analyzed using LabChart software (AD Instruments). Only high-quality ECG tracings viewed at high magnification (200 - 400%) were included in the analysis. A trained technician blinded to group allocation measured the following parameters across a minimum of three consecutive cardiac cycles: P-wave amplitude, P-wave duration, PR interval, QRS duration, QT interval, and corrected QT interval (QTc). Mean values were calculated for subsequent statistical analysis. All measurements were independently verified by a cardiac physiology specialist (Horii et al., 2025). Allocation concealment and blinding were implemented at this stage.

### 2.6 Heart rate variability analysis

Heart rate variability (HRV), defined as the beat-to-beat variation in the time intervals between successive heartbeats, was used to evaluate autonomic nervous system (ANS) activity at the exercise endpoint. The time-domain parameters included the standard deviation of all normal RR intervals (SDRRs), the root mean square of successive RR interval differences (RMSSDs), and the proportion of RR intervals exceeding 6 ms in duration relative to the preceding interval (pRR6). The frequency-domain parameters included low-frequency power (LF), high-frequency power (HF), and the LF: HF ratio.

Poincaré plot analysis was conducted on RR interval time series from 32 mice (n = 8 per group) following the methodological framework described by Figueroa et al. (2025) A total of 46,979 valid RR interval pairs were included after 347 pairs with missing values were excluded. Lag-1 Poincaré plots were constructed by plotting each RR interval (RR_i_) against the subsequent interval (RR_i+1_) and quantified using the ellipse-fitting technique to derive SD1 (short-term variability, perpendicular to the line of identity), SD2 (long-term variability, along the line of identity), ellipse area (S = π × SD1 × SD2), and the SD1/SD2 ratio. The complex correlation measure (CCM), a nonlinear descriptor based on consecutive three-point triangle areas normalized to the ellipse area, was computed to capture temporal dynamics beyond standard linear descriptors. Heart rate asymmetry (HRA) was quantified using Guzik’s index (GI), Porta’s index (PI), and Ehlers’ index (EI) to assess the directional imbalance between acceleration and deceleration events relative to the line of identity. Lagged Poincaré analysis (lag m = 1 - 10) was performed to evaluate the temporal dependency structure across multiple time scales. Three histogram projections of the Poincaré plot, RR interval, width (delta-RR), and length were generated to visualize overall, short-term, and long-term variability distributions, respectively.

### 2.7 Echocardiography

Transthoracic echocardiography was performed using a Vevo^®^ 3100 high-resolution imaging system (FUJIFILM VisualSonics, Toronto, Ontario, Canada) equipped with an MX400 ultra-high frequency linear array transducer (18–38 MHz, center transmit: 30 MHz, axial resolution: 50 μm). Anesthesia was induced by inhalation of 3% isoflurane (Baxter International, Deerfield, Illinois, USA) in 100% oxygen at a flow rate of 1 L/min, and mice were secured in the supine position on a heated platform set to 37 °C to preserve core body temperature. Following depilation of the thoracic region, pre-warmed ultrasound gel was applied to the imaging area. The isoflurane concentration was subsequently reduced to a maintenance level of 1% isoflurane to ensure stable and comparable heart rates throughout the examination. Structural and systolic functional parameters were assessed from two-dimensional guided M-mode recordings obtained from the parasternal long-axis view. Measurements included left ventricular anterior wall thickness at end-diastole (LVAWd) and end-systole (LVAWs), left ventricular posterior wall thickness at end-diastole (LVPWd) and end-systole (LVPWs), left ventricular internal dimensions at end-diastole (LVDd) and end-systole (LVDs), left ventricular end-diastolic area (LVAd) and end-systolic area (LVAs), as well as left ventricular end-diastolic volume (LVVd) and end-systolic volume (LVVs). Left ventricular mass (LV Mass) was calculated accordingly. Fractional shortening (FS) and ejection fraction (EF) were calculated. Diastolic function was evaluated using pulsed-wave Doppler from the apical four-chamber view to record peak early (E) and late (A) transmitral inflow velocities. Tissue Doppler imaging was employed to obtain early (e’) and late (a’) diastolic mitral annular velocities at the lateral wall. The E/A ratio, lateral e’/a’ ratio, and mean E/e’ ratio were calculated as established indices of diastolic function. Left atrial dimensions at end-diastole (LADd) and end-systole (LADs) were measured from M-mode tracings of the left atrium in the parasternal long-axis view. All measurements were performed by two experienced technicians blinded to group allocation and analyzed by a single blind physician (Feng et al., 2025).

### 2.8 AF inducibility measurements

Upon completion of the 10-week exercise training protocol, epicardial electrophysiology studies were performed to evaluate cardiac electrophysiological properties and susceptibility to arrhythmia. Mice were anesthetized by intraperitoneal injection of 2,2,2-tribromoethanol (Avertin; Sigma-Aldrich, St. Louis, MO, USA) at a dose of 250 mg/kg body weight. Surface electrodes were attached to the limbs to continuously monitor the surface electrocardiogram (ECG) throughout the procedure. Following a midline sternotomy to expose the heart, a multielectrode array was gently positioned on the epicardial surface of the right atrium and right ventricle. A 15-min stabilization period was allowed before initiating any electrophysiological measurements.

Multipolar electrodes were delivered to the atrial or ventricular epicardium using a programmable electrical stimulator with cycle control. Intracardiac electrograms were recorded from epicardial electrodes, and lead II surface electrocardiograms were simultaneously obtained via subdermal needle electrodes to confirm that the intracardiac A wave was aligned with the surface P wave prior to further AF induction.

For epicardial pacing, the capture threshold was determined by delivering electrical pulses at an interval 20 ms shorter than the spontaneous RR interval, with stimulus voltage progressively increased until consistent capture (i.e., each stimulus elicited a QRS complex). All pacing protocols were subsequently performed at 1.5 times the capture threshold.

The atrial effective refractory period (AERP) was measured using programmed electrical stimulation. A drive train of nine consecutive stimuli (S1) was followed by a single premature extrastimulus (S2) delivered at progressively decreasing coupling intervals. The S2 interval was initially set below the capture threshold (~15 ms) and increased in 5 ms steps until atrial capture was observed. The coupling interval was then reduced by 1–2 ms increments until capture was lost. The mean AERP for each mouse was calculated from two sequential measurements.

Atrial fibrillation (AF) inducibility was assessed by applying rapid pacing protocols to the left atrial epicardium. The stimulation protocols included: (i) 27 pulses (1 ms duration) delivered at a 40 ms interval, reduced stepwise by 2 ms decrements to 20 ms, repeated three times; and (ii) 20 pulses (1 ms duration) at a 20 ms coupling interval, repeated 20 times with 1.5 s inter-burst intervals, followed by a 5 min recovery period, repeated up to three times. Sustained arrhythmias were defined as reproducible episodes of rapid, disorganized atrial activity lasting > 3 s (Gorman et al., 2024). An animal was scored as inducible if AF was provoked in at least 3 of its 5 stimulation attempts.

All electrophysiological signals were recorded and analyzed using a high-performance multichannel electrophysiological recording and stimulation system (LEAD-7000 series, Sichuan Jinjiang Electronic Science and Technology Co., Ltd., Chengdu, China). Allocation concealment and blinding were implemented at this stage.

### 2.9 Histological assessment (H&E and Masson staining)

At the end of the exercise protocol, mice were euthanized, and hearts were rapidly excised. The left atrial tissues were carefully dissected, fixed in 4% paraformaldehyde for 24 h, embedded in paraffin, and sectioned at a thickness of 4 - 5 μm for subsequent histological staining.

Atrial sections were stained with hematoxylin and eosin (H&E) to assess general morphology and with Masson’s trichrome to evaluate interstitial fibrosis, following standard deparaffinization, rehydration, and staining protocols. Images were captured using light microscopy, and the extent of fibrosis was quantified as the percentage of blue-stained area relative to the total tissue area using ImageJ software (Niu et al., 2025). Allocation concealment and blinding were implemented at this stage.

### 2.10 Transcriptomic profiling by RNA sequencing

Total RNA was extracted from mouse atrial tissue (n = 3 per group) using TRIzol Reagent (Life Technologies, USA) following mechanical homogenization with a TissueLyser (QIAGEN, Valencia, CA). Strand-specific RNA sequencing libraries were prepared using the Invitrogen Collibri Stranded RNA Library Prep Kit (ThermoFisher Scientific) and sequenced on an Illumina NovaSeq X Plus platform by Shanghai Personalbio Technology Co., Ltd. (Shanghai, China). For each sample, raw data metrics were summarized, including total sequencing yield, the proportion of ambiguous bases, GC content, and the percentage of bases attaining Q20 and Q30 quality thresholds. Reads were quality-filtered using Fastp to remove 3′ adapter sequences and to discard reads with average quality scores below Q20, and data quality was verified by inspecting base quality, base composition, and per-read mean quality distributions. Filtered reads were aligned to the GRCm38 (mm10) mouse reference genome using HISAT2, and sequencing quality and data sufficiency were further evaluated through gene coverage uniformity, saturation analysis, and the distribution of reads across genomic regions. Gene-level read counts were quantified using HTSeq under the Union model and normalized to FPKM to permit comparison across genes and samples. Inter-sample expression correlation was assessed using the Pearson correlation coefficient to verify experimental reliability, and principal component analysis confirmed appropriate sample clustering without outliers. Differential expression analysis was performed using DESeq2 on three biologically independent samples per group, with P values adjusted for multiple comparisons by the Benjamini-Hochberg false discovery rate (FDR) method. Differentially expressed genes were defined by an adjusted P value (FDR) < 0.05 together with a |log_2_FoldChange| > 1.5, and adjusted rather than nominal P values are reported throughout.

Trend analysis based on hierarchical clustering was used to identify co-regulated gene expression modules, and Venn diagrams were constructed to determine shared DEGs across pairwise comparisons. Functional annotation was performed through GO and KEGG enrichment analyses using the clusterProfiler R package, with statistical significance assessed by the hypergeometric test. (P-value < 0.05). To assess the involvement of calcium signaling, differentially expressed genes (DEGs) were cross-referenced with calcium-regulatory genes retrieved from the GeneCards database. An initial search using the keyword “calcium regulation” yielded 14,787 genes, from which 2,291 genes meeting a relevance score threshold of ≥ 10 were retained for downstream analysis. Expected background gene frequencies for enrichment testing were derived from the Ensembl BioMart genome-wide annotation of the reference genome. Calcium signaling pathway components were mapped to the KEGG Calcium signaling pathway (map04020). All statistical analyses and visualizations were performed using R with the ggplot2 and pheatmap packages. (Yamada et al., 2025) To construct a protein-protein interaction (PPI) network underlying the high-dose exercise-associated transcriptomic response, 415 genes were curated from the RNA-seq data and assigned to six non-overlapping functional modules: calcium signaling (113 genes), cardiac remodeling (112 genes), adrenergic activation (62 genes), ECM remodeling (62 genes), cardiac contraction (42 genes), and autonomic nerve regulation (24 genes). Interaction data were retrieved from the STRING database (Mus musculus, combined score ≥ 0.9), yielding 4,226 high-confidence interaction edges. The network was visualized in Cytoscape with node color mapped to log_2_ fold change values using a continuous blue (down-regulated), white (unchanged), red (up-regulated) gradient, and separate maps were generated for each of the three comparisons (Yamada et al., 2025). Allocation concealment and blinding were implemented at this stage.

### 2.11 Optical mapping

Following the 10-week exercise training protocol, mice from all four groups were subjected to optical mapping of the heart. Mice received an intraperitoneal injection of heparin at a dose of 1000 IU/kg body weight and were subsequently anesthetized by inhalation of 5% isoflurane in 100% oxygen at a flow rate of 1 L/min. Hearts were then rapidly excised, cannulated via the aorta, and retrogradely perfused with Krebs-Henseleit (KH) solution (37 °C, gassed with 95% O_2_ and 5% CO_2_). To eliminate motion artifacts, blebbistatin (10µM; Tocris Bioscience, USA) was added to the perfusate. Hearts were then positioned in a temperature-controlled recording chamber (37 °C). For simultaneous recording of membrane voltage and intracellular Ca^2+^ transients, the hearts were dual-stained with the voltage-sensitive dye RH237 (40 µL; Abcam, UK) and the Ca^2+^-sensitive dye Rhod-2 AM (80 µL; Abcam, UK). Rhod-2 AM was initially dissolved in Pluronic acid F-127 (Abcam, UK) to facilitate membrane permeabilization and dye loading before being introduced into the perfusion line. Both dyes were delivered sequentially and allowed to equilibrate for at least 15 min before data acquisition. Optical signals were recorded from the epicardial surface of the left atrium using a high-resolution electrical imaging system (OMS-PCIE-2002, MappingLab, UK). Data acquisition was performed with OMapRecord software, and subsequent analysis was carried out with OMapScope software (Mondragon et al., 2026). Atria were continuously stimulated with S1S1 pacing at cycle lengths ranging from 180 ms to 100 ms (decrements of 20 ms). For each cycle length, we measured Ca^2+^ transient duration at 30%, 50%, and 90% repolarization (CaD_30_, CaD_50_, CaD_90_), action potential duration at corresponding repolarization levels (APD_30_, APD_50_, APD_90_), rise time and activation time of both signals, and conduction velocity (CV). At faster pacing rates (cycle lengths from 120 ms down to 60 ms, 10 ms steps), steady-state Ca^2+^ alternans was evaluated. The Ca^2+^ transient alternans ratio and diastolic Ca^2+^ level (CaD80) were quantified. A standard S1-S2 restitution protocol was employed, consisting of eight S1 stimuli (basic cycle length 150 ms) followed by a premature S2 stimulus with progressively shorter coupling intervals. For each S2, the ratio of the S2-induced Ca^2+^ transient amplitude to the last S1-induced amplitude (CaT ratio) and the coefficient of variation of the CaT ratio (COV CaT ratio) were calculated. Allocation concealment and blinding were implemented at this stage.

### 2.12 Statistical analysis

All data are expressed as Mean ± SD. The statistical analysis plan was prespecified before data unblinding. For each endpoint, normality was assessed using the Shapiro-Wilk test and homogeneity of variance using Levene’s test. If both assumptions were met, one-way ANOVA with Tukey’s *post-hoc* test was applied. If normality held but variances were unequal, Welch’s ANOVA with Tamhane’s T2 correction was used. If normality was violated, the Kruskal-Wallis test with Dunn’s *post-hoc* correction was applied. This hierarchical decision tree was applied uniformly to all endpoints and was not modified after inspection of group-level results. Specific statistical significances are indicated in the figures. Differences were considered statistically significant at *P < 0.05. All statistical analyses were performed using SPSS 27.0 and R 4.5.2. Graphical representations were generated using Origin 2024 and GraphPad Prism 10.1.2.

## 3 Results

### 3.1 Chronic treadmill exercise exerts dose-dependent effects on body weight, performance, and behavior in mice

The mean maximal tolerated speed was 23.15 m/min. On the basis of this value, three exercise dose groups were established: the low-dose group exercised at 58.32% of the maximal tolerated speed, the moderate-dose group exercised at 79.91%, and the high-dose group exercised at 99.35% (**Supplementary Figure 1**).

Observations of the mice (eight per group) before, during, and after exercise revealed differential changes in body weight, exercise performance, and behavioral outcomes across the groups. One-way analysis of variance (ANOVA) was performed at weeks 0, 2, 4, 6, 8, and 10 to evaluate changes in body weight across groups. In the low-dose group, a transient but statistically significant decrease in body weight was observed in week 2 compared with that in the control group (P < 0.05); however, no significant differences were detected at any other time point. In contrast, compared with the control group, both the moderate- and high-dose groups exhibited significant reductions in body weight beginning at week 4 of formal training. Notably, the high-dose group also showed a significant decrease in body weight as early as week 2 (P < 0.05) (**Figure 1B**).

With respect to body weight changes, the high-dose group exhibited only an 18.24% increase in body weight at the exercise endpoint relative to baseline, which was significantly lower than the 31.09% increase observed in the control group (P < 0.05). Furthermore, fitted curve analyses revealed that both food and water intake tended to decrease markedly in the high-dose group during the training period. (**Figures 1C–E**). Behavioral assessments during and after exercise sessions revealed marked differences between groups. The high-dose group had a cumulative exercise behavior score of only 22, which was substantially lower than the score of 32 in the control group, indicating poor exercise tolerance. Additionally, animals in the high-dose group displayed markedly elevated respiratory rates during exercise (P < 0.05) (**Figures 1F, G**). Finally, when cardiac weight was normalized to either body weight or tibial length, no significant differences were observed among any of the groups, indicating that the exercise interventions at the prescribed intensities and duration did not induce detectable cardiac hypertrophy.

### 3.2 Graded exercise doses differentially reshape cardiac electrophysiological properties and autonomic tone

Electrocardiographic (ECG) and heart rate variability (HRV) analyses performed on eight mice per group revealed differential changes across the groups.

**Figure 2:**
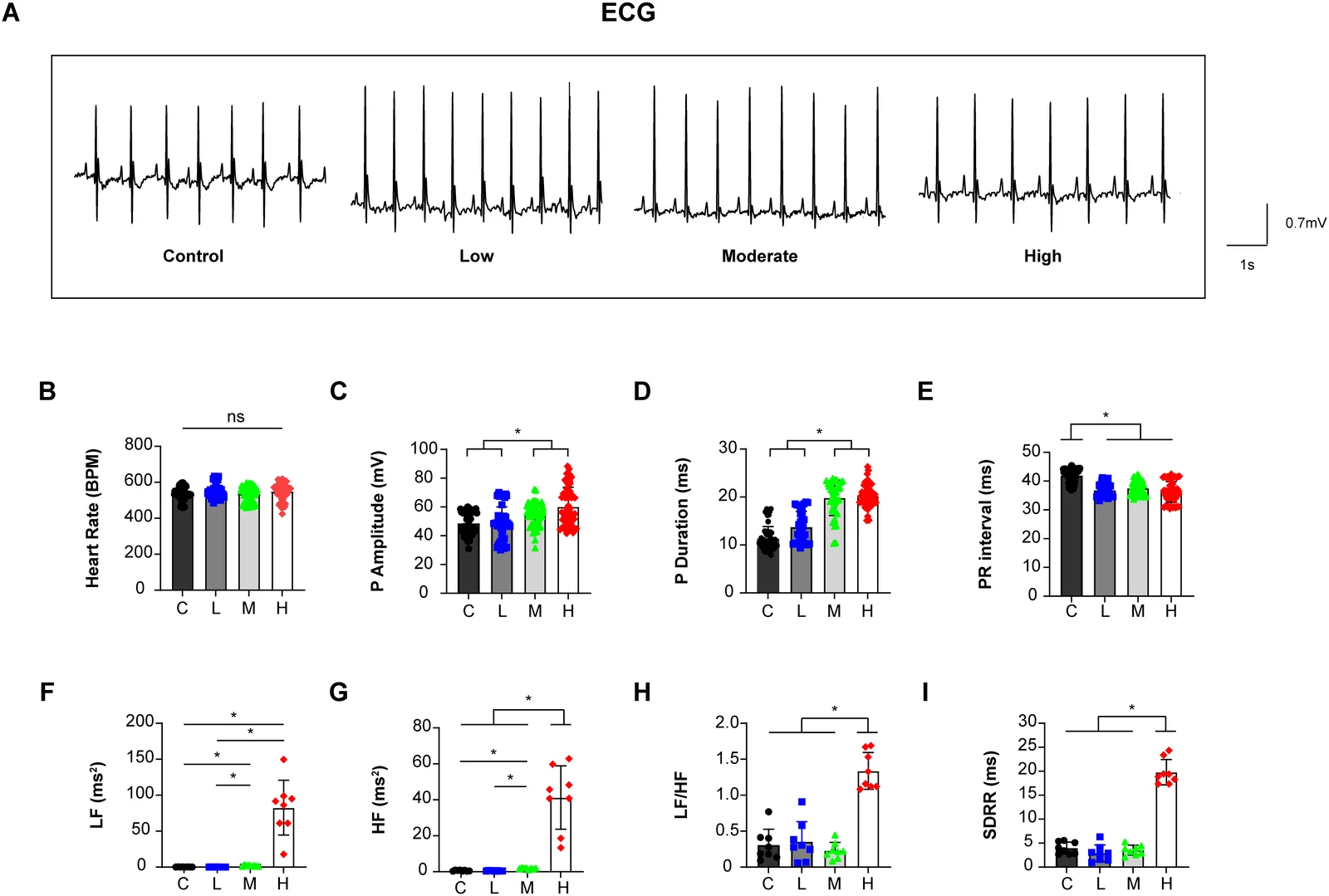
High-dose exercise induces changes in electrocardiographic parameters and heart rate variability. **(A)** Representative electrocardiogram (ECG) segments from the four experimental groups: control (C), low-dose (L), moderate-dose (M), and high-dose (H) exercise. **(B–E)** Electrocardiographic parameters. **(B)** Histograms of heart rate; each point represents the average of at least three consecutive beats (n = 8 per group). **(C)** P-wave amplitude, **(D)** P-wave duration, and **(E)** PR interval (n = 8 per group). **(F–I)** Heart rate variability (HRV) indices derived from 5-min ECG recordings. **(F)** Low-frequency (LF) power, **(G)** high-frequency (HF) power, **(H)** LF/HF ratio, and **(I)** standard deviation of RR intervals (SDRRs). Each histogram shows data from eight mice per group. Data are presented as the mean ± SD unless otherwise specified. Normality was assessed using the Shapiro-Wilk test, and homogeneity of variance was evaluated by Levene’s test. For comparisons among multiple groups, one-way ANOVA followed by Tukey’s HSD *post hoc* test was applied when both assumptions were met. When the normality assumption was violated, the Kruskal-Wallis test was used. In cases of unequal variance, Tamhane’s T2 *post hoc* test was employed. *P < 0.05.

Surface ECG analysis (**Figures 2A, B**) revealed that the P-wave amplitude (PA) (**Figure 2C**), P-wave duration (PD) (**Figure 2D**), and PR interval (**Figure 2E**) differ significantly across groups (P < 0.05 for each). Compared with that in the control and low-dose groups, the PA in the high-dose group (60.09 ± 13.53 mV) was significantly greater (P < 0.05), whereas the PD was significantly greater in both the high-dose (20.21 ± 2.28 ms) and moderate-dose (19.81 ± 3.68 ms) groups (P < 0.05). Notably, the PR interval in the control group (41.89 ± 2.28 ms) was significantly longer than that in all the exercise groups (P < 0.05). The QT and heart rate-corrected QT (QTc) intervals significantly differed across groups, whereas heart rate, QRS duration, and the RR interval did not significantly differ among groups (**Supplementary Figures 2A–D**).

**Figure 3:**
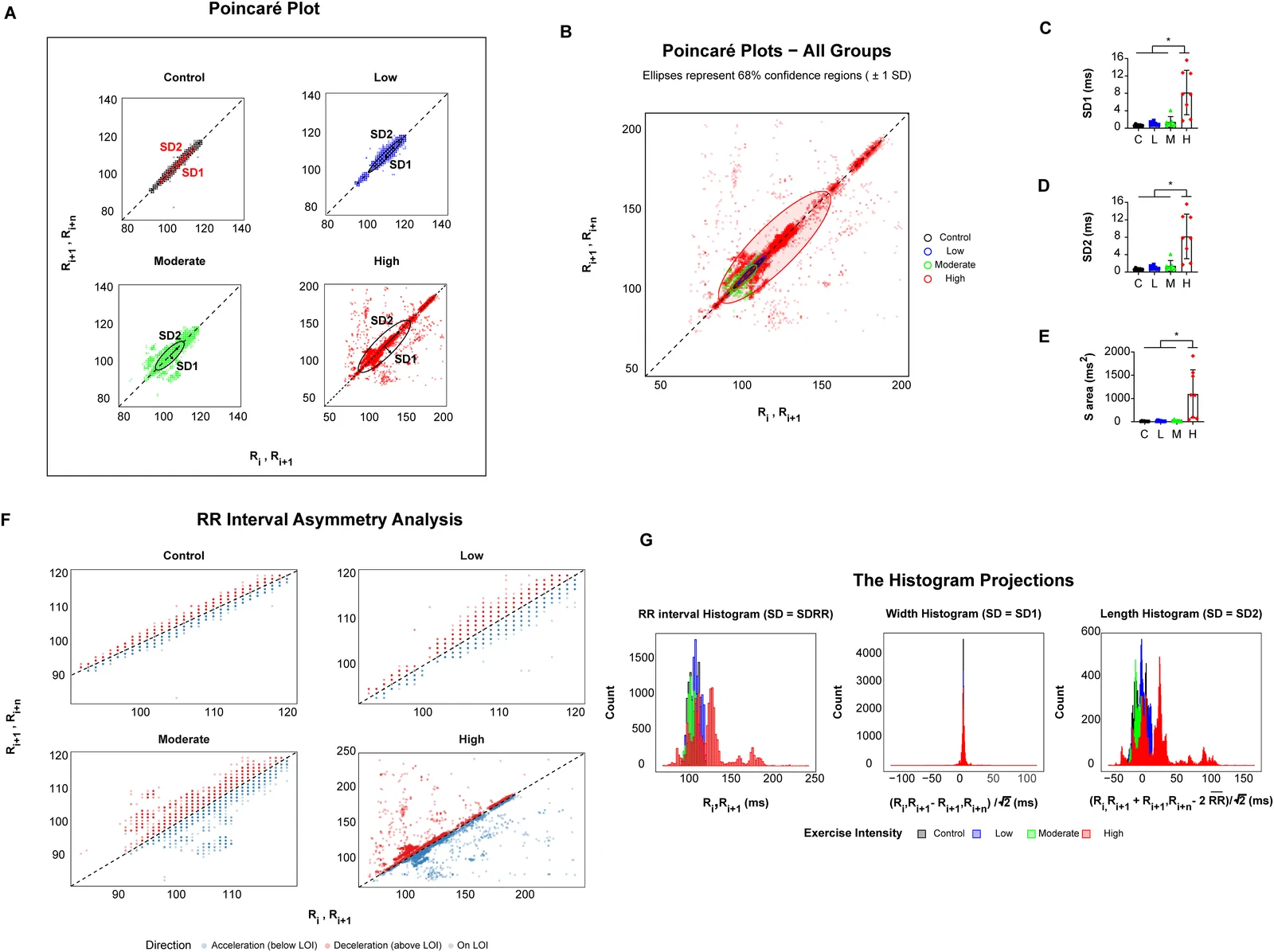
High-dose exercise disrupts heart rate variability patterns on Poincaré plot analysis. **(A)** Representative Poincaré plots for each group. The x-axis represents R_i_R_i+1_, and the y-axis represents R_i+1_R_i+n_. Arrow lengths are scaled according to the corresponding standard deviation (SD) values; the arrows indicate the orientation, and the area enclosed by the ellipse corresponds to the S area. i=1…n **(B)** Summary plot of Poincaré descriptors across all groups. **(C)** Histogram of SD1, **(D)** histogram of SD2, and **(E)** histogram of the S area. **(F)** Heart rate asymmetry (HRA) analysis: points above the line of identity (deceleration) are shown in red, and points below (acceleration) are shown in blue. **(G)** Three histogram projections derived from the Poincaré plot: RR interval histogram (projection onto the x-axis, reflecting overall HRV), width/ΔRR histogram (perpendicular to the line of identity, SD = SD1, short-term variability), and length histogram (along the line of identity, SD = SD2, long-term variability). All histograms use the count as the y-axis. Data are presented as the mean ± SD unless otherwise specified. Normality was assessed using the Shapiro-Wilk test, and homogeneity of variance was evaluated by Levene’s test. For comparisons among multiple groups, one-way ANOVA followed by Tukey’s HSD *post hoc* test was applied when both assumptions were met. When the normality assumption was violated, the Kruskal-Wallis test was used. In cases of unequal variance, Tamhane’s T2 *post hoc* test was employed. *P < 0.05.

Heart rate variability analysis demonstrated dose-dependent autonomic remodeling across both spectral and time domains. In the frequency domain (**Figures 2F–H**; **Supplementary Figures 3A, B**), low-frequency power (LF; 82.63 ± 38.06 ms²), high-frequency power (HF; 41.24 ± 17.62 ms²), the LF/HF ratio (1.34 ± 0.26), total power (TP), and very-low-frequency power (VLF) were significantly greater in the high-dose group than in all the other groups (P < 0.05 for each), indicating pronounced sympathovagal coactivation. In the time domain (**Figure 2I**; **Supplementary Figures 3C, D**), the RMSSD, SDRR, and pRR6 index were likewise significantly greater in the high-dose group than in all the other groups (P < 0.05 for each), confirming that both short-term and overall heart rate variability increased progressively with exercise dose. Poincaré plot analysis further corroborated these findings, with SD1 (8.19 ± 5.12 vs. 0.68 - 1.27 ms), SD2 (18.92 ± 9.23 vs. 4.06 - 4.44 ms), and ellipse area S (598.93 ± 515.58 vs. 9.00 - 17.44 ms²) all significantly elevated exclusively in the high-dose group compared with all the other groups (P < 0.05 for each) (**Figures 3A–E**; **Supplementary Figure 3E**). Histogram projections of the RR interval and ΔRR distributions confirmed substantially broader dispersions in the high-dose group, which was consistent with the enlarged Poincaré geometry (**Figures 3F, G**; **Supplementary Figures 3F–J**). Endurance exercise increases both sympathetic and parasympathetic tone in a dose-dependent manner, and these autonomic adaptations may evolve in parallel with the adaptive enhancement of cardiopulmonary reserve.

### 3.3 High-dose exercise induces left atrial remodeling and increases susceptibility to atrial fibrillation

To evaluate the effects of different exercise intensities on cardiac remodeling, we performed high-resolution echocardiography in mice following a controlled exercise protocol (**Table 1**; n=6 for each). The data revealed a clear dose-dependent effect on cardiac structure and function, with the most pronounced alterations observed in the high-dose exercise group, particularly those affecting left atrial dynamics.

**Table 1:** In vivo echocardiographic and haemodynamic parameters in mice.

Function	Units	Control(n=6)	Low(n=6)	Medium(n=6)	High(n=6)	P-values					
Structural		A	B	C	D	A vs B	A vs C	A vs D	B vs C	B vs D	C vs D
LVAWd	mm	0.94±0.02	0.90±0.02	0.88±0.02	0.82±0.03	1.00	0.09	<0.05	0.29	<0.05	0.85
LVAWs	mm	1.35±0.03	1.34±0.02	1.27±0.02	1.20±0.04	0.80	<0.05	<0.05	<0.05	<0.05	<0.05
LVPWd	mm	1.33±0.09	1.19±0.09	1.05±0.04	0.84±0.04	0.01	<0.05	<0.05	0.01	<0.05	<0.05
LVPWs	mm	1.28±0.09	1.21±0.05	1.06±0.03	0.94±0.03	1.00	0.07	<0.05	0.32	<0.05	0.85
LV Mass	mg	168.80±3.44	162.85±6.12	162.15±3.31	129.98±2.36	0.08	0.05	<0.05	0.99	<0.05	<0.05
Systolic											
LVDd	mm	3.55±0.11	3.82±0.07	3.90±0.08	3.94±0.09	<0.05	<0.05	<0.05	0.39	0.11	0.86
LVDs	mm	2.40±0.10	2.69±0.06	2.85±0.08	2.94±0.10	<0.05	<0.05	<0.05	0.02	<0.05	0.39
FS	%	34.24±1.21	32.58±1.62	33.39±1.38	32.65±1.73	ns	ns	ns	ns	ns	ns
LVAd	mm2	22.54±0.21	22.70±0.94	24.12±0.88	25.42±0.52	0.98	<0.05	<0.05	0.01	<0.05	0.02
LVAs	mm2	15.45±0.31	15.49±0.78	18.54±1.47	18.82±1.99	1.00	0.02	0.04	0.01	0.04	1.00
LVVd	uL	62.21±4.31	63.45±3.31	63.93±2.09	69.99±3.23	0.92	0.81	<0.05	0.99	0.01	0.02
LVVs	uL	22.80±3.02	25.49±2.52	26.30±3.50	33.32±3.00	1.00	0.85	<0.05	1.00	0.04	0.12
EF	%	65.39±1.22	64.76±1.15	67.40±1.06	63.63±1.58	ns	ns	ns	ns	ns	ns
Diastolic											
LAD	mm	5.86±0.27	6.21±0.64	6.47±0.35	6.82±0.11	ns	ns	<0.05	ns	0.03	ns
LADd	mm	1.96±0.16	1.89±0.02	1.83±0.05	1.78±0.04	0.85	0.43	0.17	0.15	<0.05	0.31
LADs	mm	1.85±0.13	1.70±0.08	1.49±0.02	1.43±0.04	1.00	0.02	<0.05	0.52	0.02	1.00
E/A	ratio	1.05±0.01	1.19±0.01	1.13±0.05	1.36±0.03	0.48	1.00	<0.05	1.00	0.18	0.37
e'/a' Lateral	ratio	1.02±0.09	1.08±0.03	1.09±0.03	0.84±0.06	ns	ns	ns	ns	ns	ns
E/e' Mean	ratio	29.24±0.48	32.25±0.92	30.26±1.71	38.82±4.47	<0.05	0.76	0.02	0.21	0.09	0.02

LVAWd : Left ventricular anterior wall thickness at end-diastole ; LVAWs : Left ventricular anterior wall thickness at end-systole ; LVPWd : Left ventricular posterior wall thickness at end-diastole; LVPWs : Left ventricular posterior wall thickness at end-systole; LV Mass : Left ventricular mass; LVDd : Left ventricular internal dimension at end-diastole; LVDs - Left ventricular internal dimension at end-systole; FS : Fractional shortening=(LVDd-LVDs)/ LVDd×100; LVAd : Left ventricular area at end-diastole; LVAs : Left ventricular area at end-systole; LVVd : Left ventricular volume at end-diastole; LVVs : Left ventricular volume at end-systole; EF : Ejection fraction=(LVVd-LVVs)/ LVVd×100; LADd : Left atrial dimension at end-diastole; LAD: Left atrial dimension; LADs : Left atrial dimension at end-systole; E/A : Ratio of early to late ventricular filling velocities; e'/a' Lateral : Ratio of early to late diastolic mitral annular velocity at lateral wall; E/e' Mean : Ratio of early transmitral flow velocity to average early diastolic mitral annular velocity. Data are presented as mean ± standard deviation. Most parameters were analyzed by one-way ANOVA; if homogeneity of variance was assumed, Tukey's post hoc test was applied, otherwise Dunnett's T3 test was used. LVAWd, FS, LVPWs, LVVs, LADs, and E/A were analyzed using the Kruskal-Wallis test (non-parametric test) with post hoc comparisons corrected by the Holm-Bonferroni method. Group comparisons: A = Control group, B = Low-intensity group, C = Moderate-intensity group, D = High-intensity group.

**Figure 4:**
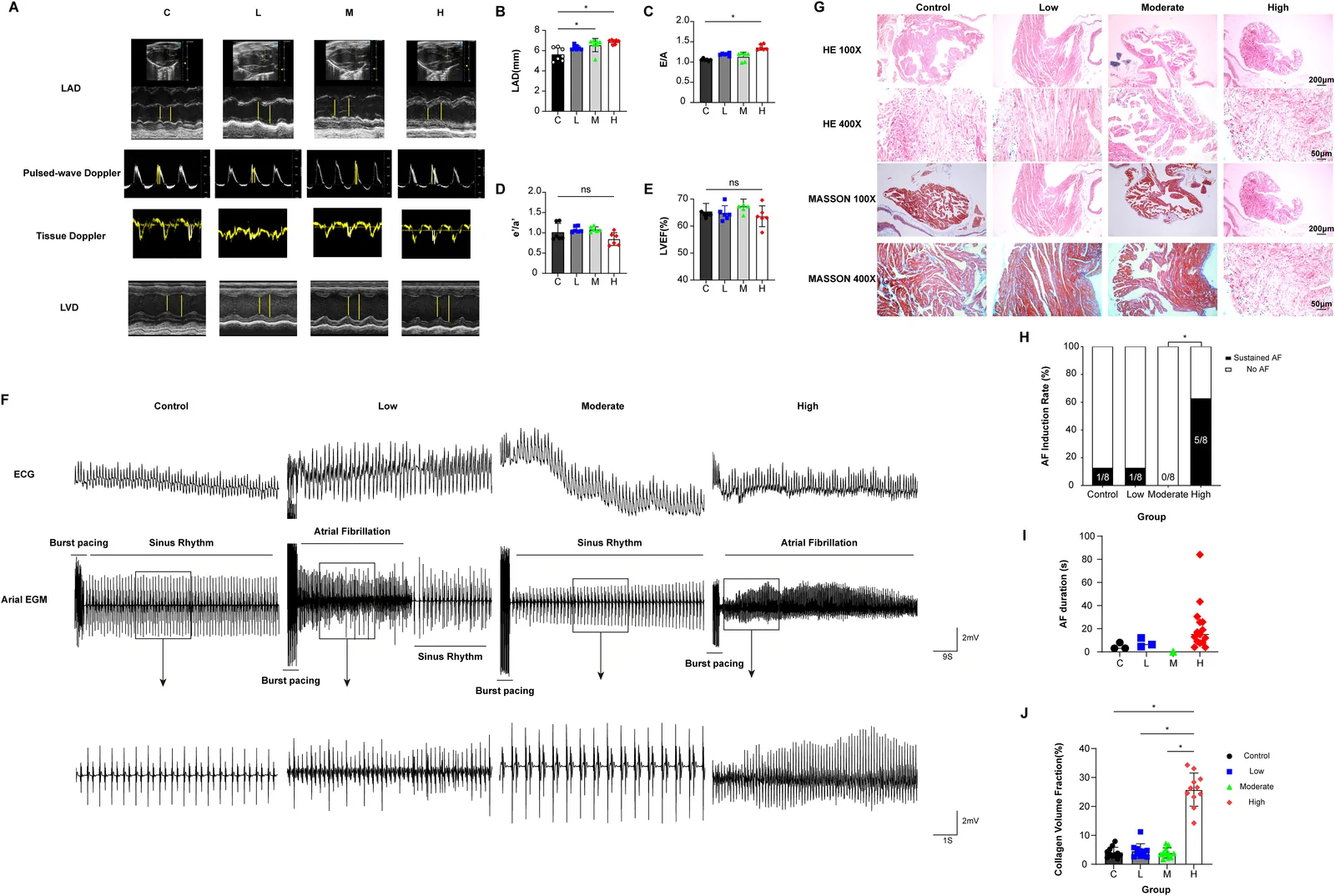
High-dose exercise induces left atrial remodeling and increases susceptibility to atrial fibrillation. **(A)** Representative echocardiographic images across experimental groups. Top to bottom: M-mode tracings of left atrial diameter; pulsed-wave Doppler of mitral inflow (E and A waves denote early rapid filling and late atrial contraction–mediated filling, respectively); tissue Doppler imaging of the lateral mitral annulus (e′ and a′ indicate myocardial velocities during early and late diastole, respectively); and M-mode tracings of left ventricular diameter. **(B–E)** Quantitative echocardiographic parameters: **(B)** left atrial dimension (LAD), **(C)** E/A ratio, **(D)** lateral e′/a′ ratio, and **(E)** left ventricular ejection fraction (LVEF). **(F)** Representative epicardial electrogram recordings. Top trace: surface limb lead ECG; middle trace: right atrial intracardiac electrogram (9 s recording); bottom trace: enlarged view of the intracardiac electrogram (1 s recording) showing detailed atrial activity. **(G)** Representative histological images of atrial sections stained with hematoxylin and eosin (H&E, top two rows) and Masson’s trichrome (bottom two rows). Magnification: 100× (first and third rows) and 400× (second and fourth rows). Scale bars: 200 μm (low magnification) and 50 μm (high magnification). **(H)** Atrial fibrillation (AF) inducibility. The percentage of episodes in which burst pacing induced AF (lasting >3 s) is shown for each group (n=8 mice per group; 5 stimulations per mouse). **(I)** Mean duration of induced AF episodes (only including mice that exhibited inducible AF in panel **G**). **(J)** Quantitative analysis of interstitial fibrosis expressed as the percentage of collagen area relative to the total tissue area from Masson’s trichrome-stained sections. The data are presented as the mean ± SD. As shown in **Figure 3H**, AF incidence was analyzed by Fisher’s exact test with Bonferroni correction for pairwise comparisons. For all other panels, one-way ANOVA followed by Tukey’s *post hoc* test or the Kruskal-Wallis test followed by Dunn’s *post hoc* test was used as appropriate. *P < 0.05.

Echocardiographic analysis revealed dose-dependent atrial structural and functional remodeling (**Figures 4A–E**). The high dose group exhibited the largest left atrial dimensions, which was significantly greater than both the control group and the moderate dose group (P < 0.05), while no statistically significant differences were observed among the control, low dose, and moderate dose groups. Consistent with these structural changes, transmitral flow assessment demonstrated a parallel dose-dependent elevation in the E/A ratio (high-dose: 1.36 ± 0.03 vs. control: 1.05 ± 0.01; P < 0.05), indicating progressive impairment of the contribution of the atrium to ventricular filling. The E/e’ ratio, a surrogate for left ventricular filling pressure, was likewise significantly elevated in the high-dose group compared with that in both the control group and the moderate-dose group (P < 0.05). Notably, despite these diastolic alterations, left ventricular systolic function-as assessed by ejection fraction and fractional shortening-remained unchanged across all groups, indicating that high-dose exercise selectively impaired atrial structure and diastolic filling dynamics while preserving global ventricular contractile performance.

To determine whether exercise dose affects AF susceptibility, AF inducibility was assessed as the proportion of animals in which sustained AF could be provoked. The AF induction rates were 12.5% (1/8) in the control group, 12.5% (1/8) in the low-dose exercise group, 0% (0/8) in the moderate-dose exercise group, and 62.5% (5/8) in the high-dose exercise group (**Figures 4F, H**). Among the animals in which AF was successfully induced, we quantified the duration of each episode to further characterize the severity of the arrhythmia (**Figure 4I**). AF episodes in the high-dose group showed markedly greater variability and duration than those in the other groups did, with individual episodes ranging from a few seconds to approximately 84 s and a median duration of approximately 15 s. In contrast, AF episodes recorded in the control and low-dose groups were generally brief (approximately 4–9 s and 6–15 s, respectively), whereas no episode was detected in the moderate-dose exercise group. The substantially longer and more variable AF episodes in the high-dose exercise group suggest that high-dose exercise not only increased AF inducibility but also reflected a more sustained proarrhythmic substrate.

In terms of pathology, compared with the control group, the high-dose exercise group exhibited markedly widened interstitial spaces, which are indicative of fibrosis, inflammatory cell infiltration, disorganization of myocardial fiber bundles, and overall reduced staining dose (**Figure 4G**). We further quantified atrial fibrosis by measuring the collagen volume fraction (CVF) using Masson’s trichrome staining (**Figure 4J**; **Supplementary Figures 4A–D**). CVF was markedly elevated in the high-dose exercise group (approximately 26 ± 6%), whereas it remained low and comparable across the control (approximately 5 ± 1%), low-dose exercise (approximately 6 ± 2%), and moderate-dose exercise (approximately 4 ± 1%) groups. The CVF in the high-dose exercise group was significantly greater than that in the other groups (all P < 0.05). These histological findings directly demonstrate atrial fibrosis and inflammatory remodeling at the tissue level.

### 3.4 Dysregulated atrial Ca^2+^ signaling may contribute to high-dose exercise-induced atrial remodeling

**Figure 5:**
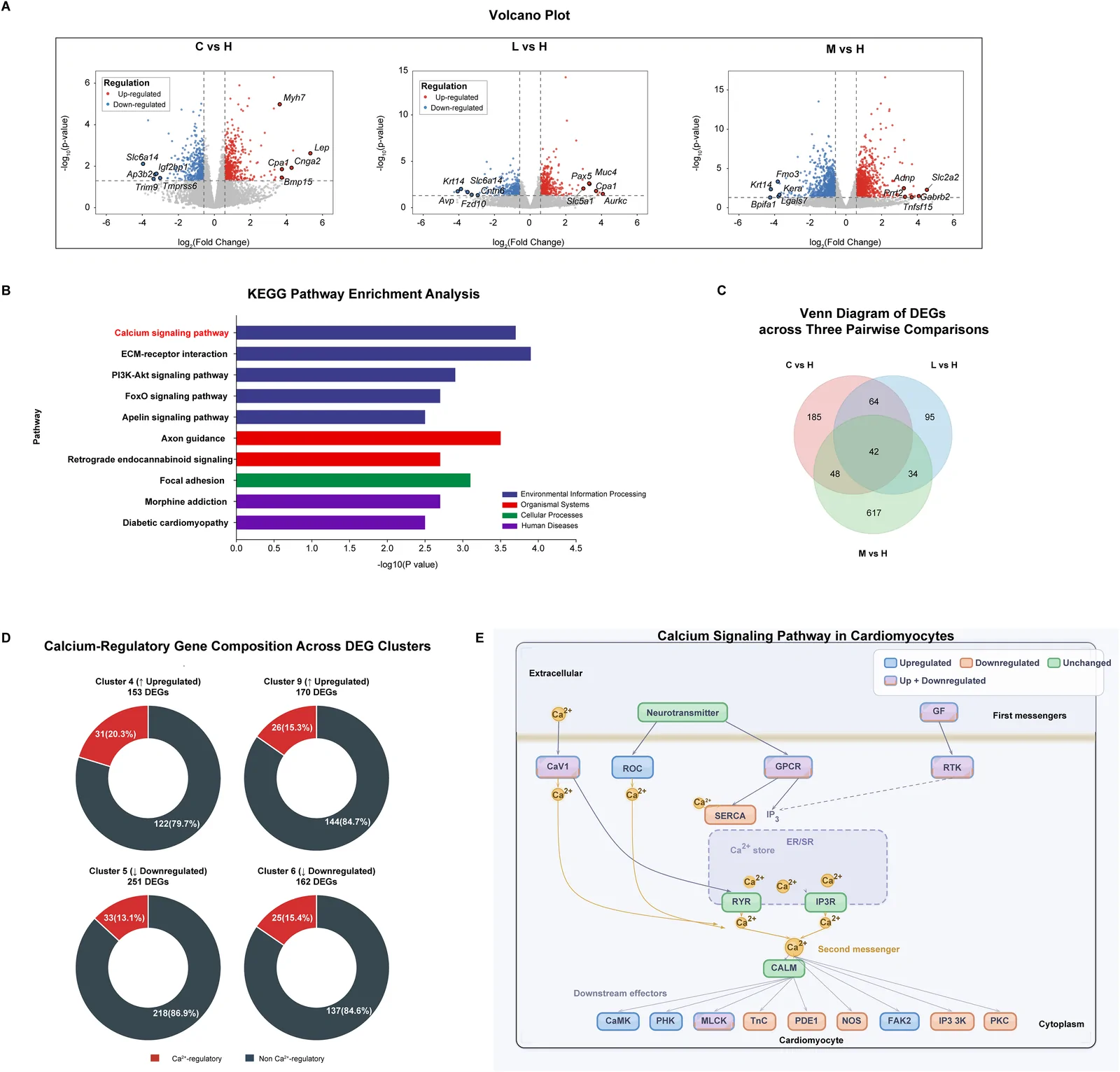
High-dose exercise induces bidirectional remodeling of the atrial calcium signaling transcriptome in mice. **(A)** Volcano plots depicting DEGs in the high-dose group compared with the control (left), low-dose (middle), and moderate-dose (right) groups. Red, up-regulated; blue, down-regulated; gray, not significant. Dashed lines indicate thresholds of |fold change| ≥ 1.5 and P < 0.05. Larger points with black borders denote calcium-regulation-associated genes, with the top five up-regulated and top five down-regulated genes labeled in each plot. **(B)** KEGG pathway enrichment analysis showing the top 10 pathways grouped by Environmental Information Processing (blue), Organismal Systems (red), Cellular Processes (green), and Human Diseases (purple). **(C)** Venn diagram of DEG overlaps among the three pairwise comparisons. Numbers indicate shared and unique gene counts; 42 genes were common compared to all three comparisons. **(D)** Donut charts showing the proportion of Ca^2+^-regulatory (red) versus non-Ca^2+^-regulatory (dark blue) genes within four differentially expressed gene (DEG) clusters identified by RNA sequencing. Upper panels represent up-regulated clusters (Cluster 4, 153 DEGs; Cluster 9, 170 DEGs), and lower panels represent down-regulated clusters (Cluster 5, 251 DEGs; Cluster 6, 162 DEGs). Percentages denote the fractional contribution of each gene category within the respective cluster. **(E)** Schematic of calcium signaling pathway alterations in atrial cardiomyocytes. Red, up-regulated; blue, down-regulated; green, unchanged; purple, bidirectionally regulated.

RNA sequencing of atrial tissue revealed substantial transcriptomic differences when comparing the high-dose group with each of the other groups (fold change ≥ 1.5, P < 0.05). Compared with the control group, 947 differentially expressed genes (DEGs) were identified, comprising 556 up-regulated and 391 down-regulated genes; compared with the low-dose group, 758 DEGs were detected (458 up-regulated and 300 down-regulated); and compared with the moderate-dose group, 2,237 DEGs were identified (1,169 up-regulated and 1,068 down-regulated) (**Figure 5A**). Cross-referencing against a comprehensive GeneCards calcium-regulation gene set revealed that 163 (17.2%), 120 (15.8%), and 305 (13.6%) of the DEGs in the three comparisons, respectively, were functionally annotated to calcium-regulatory processes, indicating broad and bidirectional remodeling of the atrial calcium-handling transcriptome specifically associated with high-dose exercise.

KEGG pathway analysis revealed enrichment of the calcium signaling pathway, suggesting a potential association between calcium signaling perturbation and high-dose exercise-induced atrial remodeling at the transcriptomic level (**Figures 5B, E**). Among the 42 DEGs commonly dysregulated across all three comparisons, 8 (19.0%) were related to calcium regulation, a proportion consistent with the involvement of calcium-related pathways in this remodeling phenotype (**Figure 5C**).

To determine whether calcium regulatory genes exhibited a similar dose-dependent trend, a cluster analysis was performed. The expression patterns of four clusters significantly increased in dose-dependent manner: Clusters 4 and 9 were markedly upregulated, whereas Clusters 5 and 6 were markedly downregulated in the high-dose group compared with those in the control group (**Figure 5D**). Across the four differentially expressed clusters, the proportions of calcium-regulatory genes were 20.3%, 15.3%, 13.1%, and 15.4%. This pervasive enrichment across both upregulated and downregulated expression programs indicates that calcium signaling disruption is not confined to a single directional change but rather represents a bidirectional feature of the transcriptional reprogramming induced by high-dose exercise.

**Figure 6:**
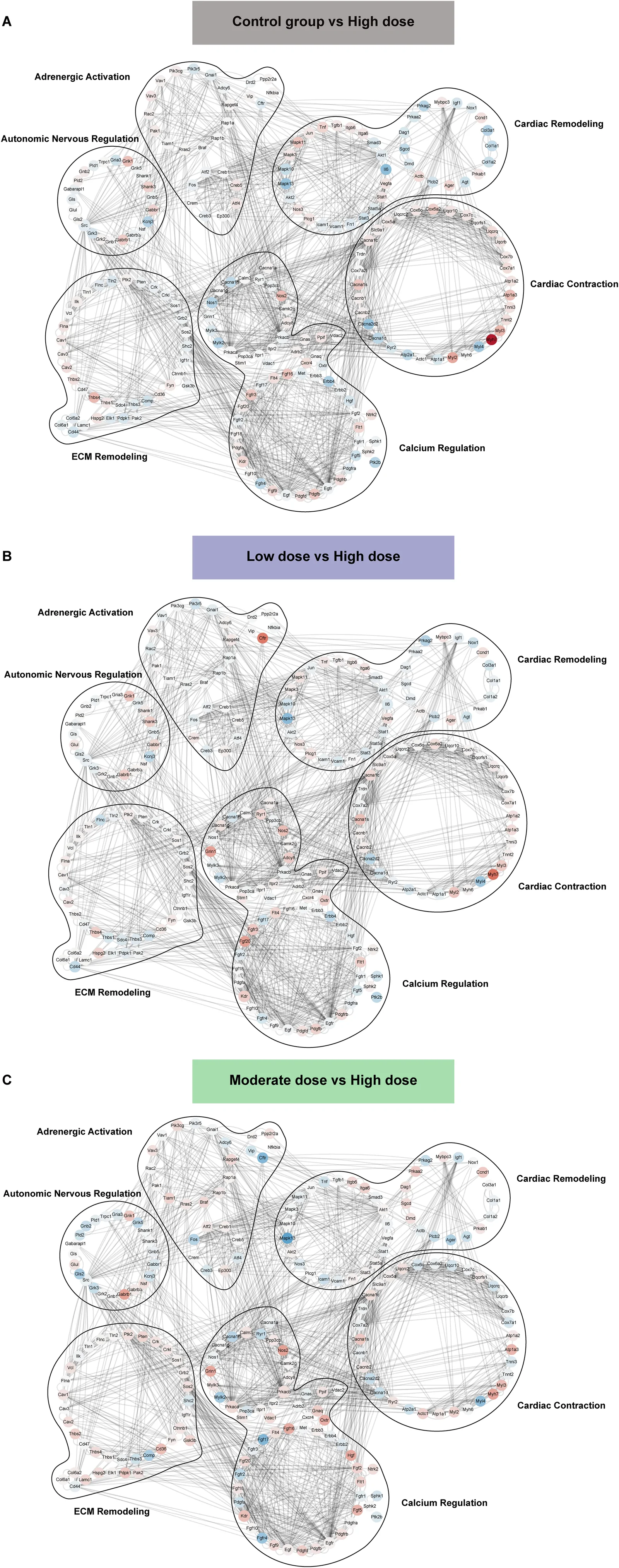
Protein-protein interaction network of exercise dose-dependent transcriptomic alterations in the atrium. **(A–C)** PPI networks constructed from 415 candidate genes comparing the high-dose group with the control **(A)**, low-dose **(B)**, and moderate-dose **(C)** groups. Interaction data were retrieved from the STRING database (Mus musculus, combined score ≥ 0.9), yielding 4,226 high-confidence edges. Nodes are colored according to differential expression direction: red, up-regulated; blue, down-regulated; gray, not significantly regulated, with color dose scaled to |log_2_ fold change|. Node size is proportional to degree centrality, and edge thickness corresponds to the STRING combined confidence score. Dashed ellipses delineate six non-overlapping functional modules: calcium regulation, cardiac contraction, cardiac remodeling, adrenergic activation, autonomic nerve regulation, and ECM remodeling.

PPI network analysis (**Figures 6A–C**) of 415 candidate genes organized into six non-overlapping functional modules, namely calcium signaling (113 genes), cardiac remodeling (112 genes), adrenergic activation (62 genes), ECM remodeling (62 genes), cardiac contraction (42 genes), and autonomic nerve regulation (24 genes), yielded 4,226 high-confidence interaction edges (STRING combined score ≥ 0.9). The calcium signaling and cardiac remodeling modules, which collectively comprised more than half of all network nodes (225/415), displayed the highest intra-modular connectivity and functioned as central hubs bridging inter-modular interactions. Notably, the differential expression patterns remained largely consistent across all three comparisons, with the majority of nodes exhibiting concordant directionality irrespective of the reference group, thereby reinforcing the specificity of the transcriptomic signature associated with high-dose exercise. Together, these results demonstrate that high-dose exercise induces coordinated transcriptional reprogramming across functionally interconnected calcium-handling, contractile, adrenergic, autonomic, and structural remodeling networks within the atrium.

### 3.5 High-dose exercise exacerbates abnormalities in Ca^2+^ handling ability and electrophysiological instability

Optical mapping experiments in the mice revealed that high-dose exercise exacerbates abnormalities in Ca^2+^ handling and aggravates electrophysiological instability.

**Figure 7:**
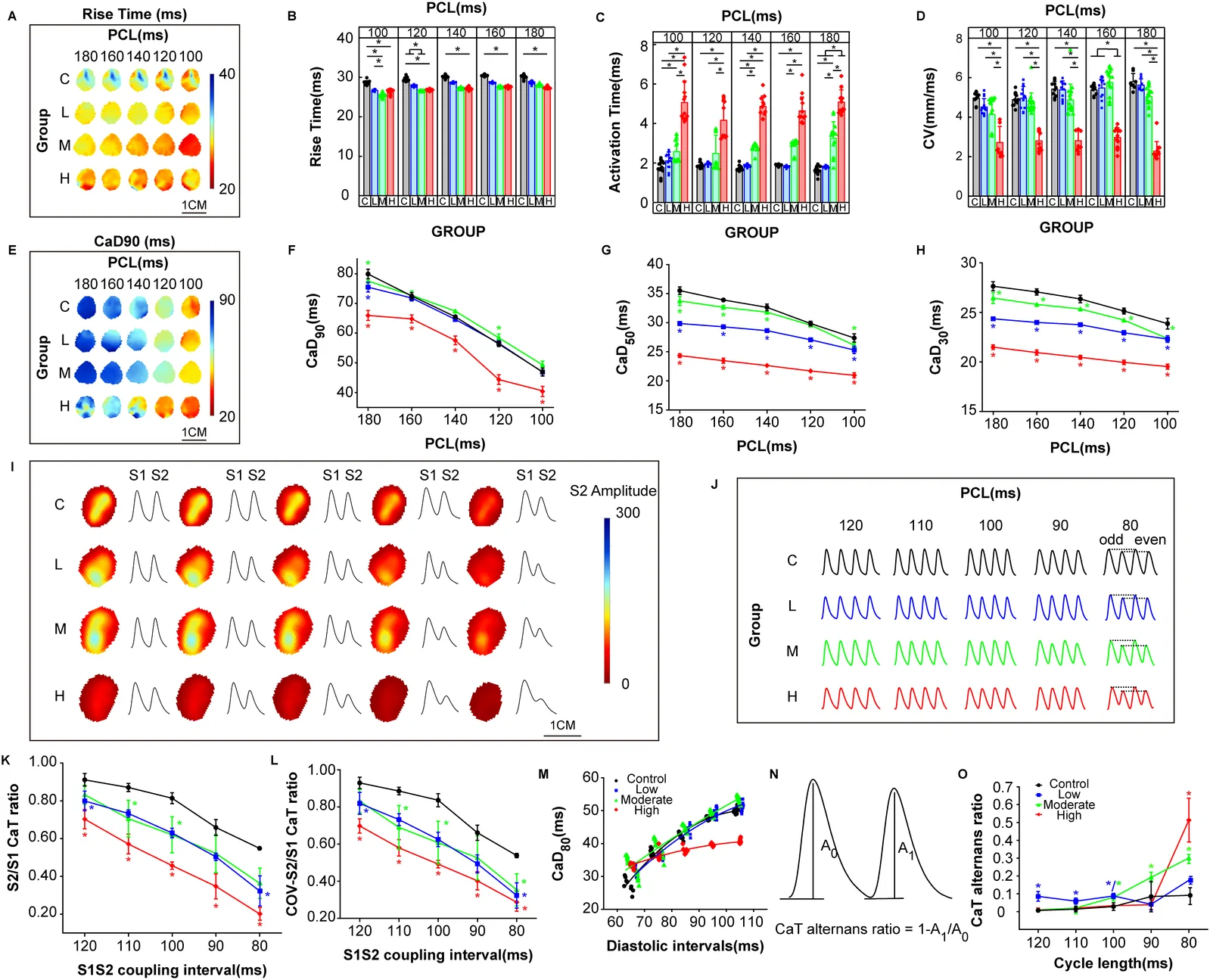
High-dose exercise promotes abnormal Ca^2+^ handling ability. **(A)** Heatmap of calcium transient rise time across pacing cycle lengths for the four groups. The color scale represents the normalized rise time (blue: prolonged; red: shortened). **(B)** Bar graph of the rise time at each pacing cycle length. **(C)** Bar graph of activation time. **(D)** Bar graph of the conduction velocity (CV). **(E)** Heatmap showing CaD_90_ values across five pacing cycle lengths (100–180 ms) for the four groups (Control, Low-dose, Moderate-dose, and High-dose). The color scale represents normalized CaD_90_ (blue: prolonged; red: shortened). **(F)** Line plot of CaD_90_ as a function of pacing cycle length. **(G, H)** Corresponding line plots for CaD_50_ **(G)** and CaD_30_ **(H)**. **(I)** Representative traces illustrating the amplitude of the S2-evoked Ca^2+^ transient and the superimposed waveforms of the last S1 and the S2 beat. **(J)** Representative calcium transient alternans traces during S1S1 pacing at decreasing cycle lengths. Traces 1 and 3 represent the first beat, and traces 2 and 4 represent the second beat. **(K)** Line plot of the CaT ratio (amplitude of the S2-evoked Ca^2+^ transient normalized to the last S1-evoked amplitude) as a function of the S1-S2 coupling interval. **(L)** Line plot of the coefficient of variation (COV) of the CaT ratio across coupling intervals. **(M)** Diastolic calcium level at 80% recovery (CaD_80_) plotted against the diastolic interval (DI). Polynomial fitting was applied to generate the restitution curves. **(N)** Schematic illustration of the calculation method for the Ca^2+^ transient alternans ratio. **(O)** Line plot of the Ca^2+^ transient alternans ratio across pacing cycle lengths from 120 to 80 ms. All quantitative data are presented as the mean ± SD. Statistical comparisons were performed using one-way ANOVA or the Kruskal-Wallis test followed by appropriate *post hoc* pairwise comparisons (see Methods). Significance indicators: *p < 0.05.

Analysis of calcium transient propagation dynamics revealed distinct dose-dependent patterns for each parameter across five pacing cycle lengths (100–180 ms). The calcium transient rise time (RT), reflecting the time to peak Ca^2+^ amplitude, decreased progressively with increasing exercise dose, with all four groups forming distinct subsets at slower pacing rates (all P <0.05); however, a ceiling effect emerged at faster frequencies, where the moderate- and high-dose groups converged while the control group remained significantly separated from all the exercise groups (P < 0.05), indicating that exercise-induced RT decrease peaks at moderate dose (**Figures 7A, B**). Activation time (AT), reflecting the Ca^2+^ excitation process, exhibited an opposing directional response: the high-dose group displayed consistently prolonged AT at every pacing frequency (all P <0.05), whereas low-dose training had a minimal effect and moderate-dose training occupied an intermediate position more clearly at slower rates (**Figure 7C**). In contrast to the graded dose-response patterns of RT and AT, the conduction velocity (CV) exhibited a threshold-type response: the CV was reduced exclusively in the high-dose group, with no significant differences among the control, low-, and moderate-dose groups at any frequency (high vs. all others P < 0.05). Moreover, the CV remained relatively stable across pacing frequencies without the pronounced rate-dependent gradients observed for RT (**Figure 7D**). The concurrent prolongation of AT and reduction in CV, observed predominantly in the high-dose group, may reflect a dual electrophysiological substrate characterized by prolonged activation latency and diminished propagation velocity. These findings suggest that high-intensity training is potentially associated with conduction-related remodeling that could, in turn, favor an arrhythmogenic substrate in atrial tissue.

Analysis of calcium transient duration at 90%, 50%, and 30% recovery levels (CaD_90_, CaD_50_, and CaD_30_, respectively) across five pacing cycle lengths (100–180 ms) revealed consistent dose-dependent shortening with increasing exercise dose (**Figures 7E–H**). At all pacing frequencies and repolarization levels, CaD followed a rank order: control > moderate > low > high dose (all P < 0.05), with the high-dose group significantly separated from all the other groups at virtually every comparison. A key finding was the progressive widening of between-group differences as the pacing rate decreased: for CaD_90_, the mean control-versus-high-dose difference expanded from 6.38 ms at 100 ms pacing to 13.97 ms at 180 ms pacing, with similar trends for CaD_50_ and CaD_30_. This frequency-dependent amplification indicates that exercise-induced adaptations in Ca^2+^ reuptake and sequestration are most fully expressed under conditions of longer diastolic intervals, when sufficient time is available for complete Ca^2+^ cycling between beats. Notably, compared with the controls, the moderate-dose group showed no significant difference in CaD_90_ from controls at faster frequencies but showed significantly shorter CaD_90_ at slower frequencies, suggesting that moderate training-induced changes in Ca^2+^ handling emerge only when the diastolic filling time is adequate to reveal the underlying remodeling.

Under premature stimulation, analysis of Ca^2+^ restitution kinetics revealed progressive, dose-dependent impairment of diastolic calcium recovery. Both the CaT ratio and its coefficient of variation (COV) decreased monotonically as the coupling interval shortened from 120 to 80 ms across all groups, with significant between-group differences at every interval (all P < 0.05; **Figures 7I, K, L**). A consistent hierarchical pattern emerged: the control group maintained the highest and most stable S2-evoked CaT recovery, the high-dose group exhibited the most severe rate-dependent depression, and the low- and moderate-dose groups occupied intermediate positions. Between-group divergence was most pronounced at the shortest coupling interval (80 ms), where the control group differed significantly from all the exercise groups; however, the low-, moderate-, and high-dose groups converged at this interval, suggesting that maximal premature stress reveals a common threshold of Ca^2+^ handling dysfunction regardless of training dose These findings suggest that, among the groups examined, high-dose exercise was associated with a comparatively greater degree of impairment in SR Ca^2+^ restitution across the range of premature coupling intervals tested, pointing to a potential dose-dependent effect on intracellular calcium handling.

Calcium alternans is a hallmark of impaired Ca^2+^ handling that promotes action potential duration alternans and arrhythmogenesis (**Figure 7J**). We further characterized this phenomenon by analyzing the diastolic calcium recovery ratio as a function of the diastolic interval (DI). In all groups, CaD_80_ decreased monotonically with decreasing DI; however, group divergence became apparent below 100 ms DI (**Figure 7M**). The control, low-, and moderate-dose groups were largely superimposable, exhibiting high CaD_80_-DI restitution slope values with preserved DI-dependent modulation, indicative of intact beat-to-beat Ca^2+^ handling. By contrast, the high-dose group exhibited the lowest restitution slope magnitude: CaD_80_ was suppressed even at long DIs, indicating impaired SR Ca^2+^ resequestration capacity independent of available diastolic time, and remained unresponsive to further DI reduction at short DIs, such that the normal DI-dependent gradient in CaD_80_ was largely abolished. The calcium alternans ratio (**Figures 7N, O**) was further employed to quantify the magnitude of beat-to-beat Ca^2+^ transient alternation across pacing intervals. The observed pattern is consistent with the notion that impaired diastolic Ca^2+^ recovery may contribute to alternans onset and could be associated with a dose-dependent increase in arrhythmogenic susceptibility.

On the depolarization and repolarization phases of the action potential, we observed signal alterations analogous to those described above for the calcium release and calcium reuptake phases of the calcium transient. Within the PCL range of 120–180 ms (**Supplementary Figures 7A, B**), rise time was significantly shortened in the high-dose group (P < 0.05). Across the full range of pacing cycle lengths examined, activation time (AT) was approximately 2- to 2.5-fold prolonged and conduction velocity (CV) was markedly reduced in the high-dose group relative to all other groups (P < 0.05), while the control, low-dose, and moderate-dose groups remained statistically indistinguishable at most pacing frequencies (**Supplementary Figures 7C, D**). Action potential duration (APD), assessed across all three repolarization levels (APD_90_, APD_50_, and APD_30_), followed a distinctly different, graded pattern (**Supplementary Figures 7E–H**). At slower pacing rates (PCL 140–180 ms), the four groups segregated into well-separated, internally homogeneous clusters, with APD shortening scaling progressively and consistently with exercise dose. As pacing frequency increased, however, the moderate- and high-dose groups progressively converged, and statistically discernible intergroup differences were no longer detectable at the fastest pacing rate examined (PCL 100 ms).

## 4 Discussion

A noteworthy feature of our forced treadmill paradigm is that high-dose endurance training in rodents elicits not only cardiopulmonary adaptation but also broad systemic responses spanning the sensory, neuroimmune, and endocrine axes. In the present study, the physiological and behavioral deterioration observed in the high-dose group, including reduced weight gain, diminished food and water intake, elevated respiratory rate, prolonged recovery, decreased locomotion, impaired motor coordination, deteriorated fur quality, and abnormal ocular condition, appeared to reflect the chronic inflammatory burden and systemic stress imposed by repetitive exhaustive exercise (Murugathasan et al., 2023). These observations suggest that the high-dose phenotype likely reflects the integrated physiological burden of sustained high-intensity exercise, in which sympathetic hyperactivation, neuroendocrine perturbation, and systemic inflammation are inherent components rather than separable confounders. The concurrent autonomic imbalance and behavioral alterations observed in the high-dose group may therefore represent coordinated manifestations of this cumulative allostatic load, potentially involving tryptophan-kynurenine pathway-mediated neuroimmune crosstalk (Yamashita, 2020).

Second, only male C57BL/6 mice were enrolled. Although this design minimized estrous cycle-related variability, murine atrial electrophysiology differs substantially between sexes. Regarding repolarization, male mice exhibit approximately 25% greater ventricular K_v_ current density and 8-fold higher KCNE4 expression, with Kcne4 deletion reducing atrial K_v_ currents by >45% in males (Crump et al., 2016). Female mice display a 14% longer atrial effective refractory period than males (Obergassel et al., 2021). Concerning conduction and Ca^2+^ handling, male atrial myocytes show greater connexin 40/43 lateralization, a 28% larger Ca^2+^ transient amplitude, 54% higher NCX1 current density, and more frequent spontaneous Ca^2+^ releases (Thibault et al., 2022b; Thibault et al., 2022a). Upon programmed electrical stimulation, males exhibit approximately 50% higher AF inducibility, a difference abolished by orchiectomy (Thibault et al., 2022b). In the present study, all electrophysiological assessments were derived exclusively from male animals; accordingly, the contribution of sex to the observed atrial remodeling and AF vulnerability cannot be addressed, and future studies incorporating both sexes are warranted.

Our findings suggest that high-dose exercise training increases AF susceptibility in male mice by concurrently disrupting atrial electrical stability and structural integrity. Electrocardiographically, the high-dose group exhibited elevated P-wave amplitude, prolonged P-wave duration, and shortened PR interval, accompanied by sympathovagal coactivation across time-domain, frequency-domain, and nonlinear HRV measures (Cavigli et al., 2022). This autonomic perturbation may directly participate in atrial electrical remodeling through the intrinsic coupling between neurotransmitter signaling and ion channel function (Shen and Zipes, 2014; Huang et al., 2025). The dose-dependent nature of these changes suggests that autonomic remodeling is not merely a bystander phenomenon but also an active contributor to the dose-dependent arrhythmogenic substrate.

At the organ and tissue levels, the high-dose group displayed echocardiographic evidence of atrial remodeling alongside histopathological findings of inflammatory cell infiltration and pronounced atrial myocardial fibrosis, observations consistent with established models of exercise-induced atrial arrhythmogenic remodeling (Aschar-Sobbi et al., 2015; Meza-Ramos et al., 2023; Dorian et al., 2024; McHugh et al., 2026). Moreover, programmed electrical stimulation markedly elevated AF inducibility and increased arrhythmic burden in the high-dose group, whereas the moderate-dose group exhibited a complete absence of inducible AF. This dichotomy further reinforces the U-shaped relationship between exercise dose and AF risk (Franklin et al., 2022), indicating that moderate-dose exercise remains within the window of adaptive remodeling, while high-dose exercise exceeds the threshold for maladaptive transformation. Taken together, high-dose exercise simultaneously operates at two distinct but converging levels: systemically, it shifts the autonomic tone toward a proarrhythmic sympathovagal profile, whereas locally, it drives maladaptive atrial remodeling characterized by fibrosis, inflammation, and electrophysiological substrate formation. The convergence of these systemic neural perturbations and focal organ-level structural alterations collectively furnishes a vulnerable substrate for both the initiation and maintenance of atrial fibrillation.

A central and novel finding of the present study is that high-dose endurance exercise destabilizes atrial calcium regulatory signaling in a dose-dependent manner. Exercise-induced atrial structural remodeling, encompassing fibrosis, inflammatory infiltration, and geometric alteration, has been associated with progressive impairment of atrial mechanical and electrical function. Notably, calcium signaling dysregulation may not be limited to a downstream consequence of this remodeling cascade but could also represent an integral component intertwined with the mechanoelectrical coupling process itself (Quinn and Kohl, 2021; Medvedev et al., 2024). Specifically, structural alterations are thought to impose aberrant mechanical loading and attenuate intercellular coupling across the atrial syncytium, which may in turn perturb intracellular calcium homeostasis through activation of stretch-sensitive ion channels (Buonocunto et al., 2024), CaMKII-dependent hyperphosphorylation of RyR2, and impaired sarcoplasmic reticulum calcium cycling (Heijman et al., 2020). Such calcium signaling perturbation could subsequently feed forward to further destabilize electrical properties, potentially shortening action potential duration, facilitating delayed afterdepolarizations, and amplifying conduction heterogeneity (Heijman et al., 2014). Thus, the co-occurrence of aberrant Ca^2+^ signal transduction with atrial structural remodeling and an arrhythmogenic electrophysiological substrate observed in the present study suggests a potential intrinsic association among these pathological alterations.

Having observed transcriptomic signatures suggestive of calcium signaling dysregulation following high-dose exercise, we next examined whether these molecular-level perturbations might be accompanied by detectable alterations in macroscopic atrial calcium handling. Optical mapping of calcium transients in intact atrial preparations, performed under blebbistatin to suppress motion artifacts (Enriquez-Vazquez et al., 2023), revealed that high-dose exercise tended to be associated with calcium handling perturbations across four domains: altered transient onset, abbreviated transient recovery, divergent frequency-dependent responses, and impaired diastolic calcium dynamics.

Calcium transient duration progressively shortened with increasing exercise dose, and intergroup separation widened at slower pacing rates, a pattern that may reflect the unmasking of intrinsic differences in LTCC gating kinetics, SR calcium load, and SERCA2a reuptake efficiency during prolonged diastolic intervals (Baines et al., 2024). Decomposition of the calcium transient upstroke further revealed a dissociation between accelerated rise time and prolonged activation time in the high dose group, a finding compatible with augmented SR calcium release coexisting with subtly impaired wave initiation, possibly arising from reduced LTCC RyR2 coupling fidelity (Medvedev et al., 2024; Miotto et al., 2024,) whereas conduction velocity exhibited a threshold type reduction largely confined to this group, consistent with, though not exclusively attributable to, interstitial fibrosis imposing physical barriers to intercellular propagation. Further analysis of diastolic Ca^2+^ restitution revealed a perturbed recovery-release equilibrium in the high-dose group, manifesting as a markedly reduced restitution slope and attenuated Ca^2+^ recovery at short diastolic intervals, observations that may reflect impaired SERCA2a mediated reuptake and elevated diastolic SR calcium leakage through dysfunctional RyR2 (Steinberg et al., 2023), potentially heightening the propensity for calcium transient alternans at faster pacing rates and blunting premature beat calcium responsiveness (Liang et al., 2024). The action potential duration and the calcium reuptake process exhibited concordant alterations, manifesting as a reduction in the parametric concordance between CaTD and APD in the high-dose group across all pacing cycle lengths examined, in keeping with the well-recognized interdependence between calcium handling and electrical repolarization in atrial tissue (Cameron et al., 2025). Taken together, these tissue-level observations are consistent with the notion that transcriptomic perturbations in calcium regulatory genes may coincide with subtle alterations in calcium wave initiation, propagation, recovery, and cycling stability within atrial cardiomyocytes, and that such alterations, in concert with concurrent structural remodeling, could potentially contribute to a substrate permissive to arrhythmogenesis in the setting of exercise-induced atrial fibrillation.

While the aforementioned findings shed light on tissue-level perturbations in atrial calcium signaling, they warrant cautious interpretation in the context of inherent methodological constraints. One such constraint arises from the necessity of excitation-contraction uncoupling during optical mapping. Although blebbistatin (10 µM) completely abolishes contraction without altering action potential morphology, conduction, or Ca^2+^ transient kinetics (Fedorov et al., 2007), it is not entirely inert: secondary effects on electrical activity, metabolic demand, and coronary flow have been documented, and improper administration may lead to erroneous interpretation of optical signals (Swift et al., 2021). The pronounced structural remodeling in the high-dose group theoretically raises differential susceptibility to such perturbation. However, identical drug concentrations and perfusion protocols were applied across all groups, and our conclusions rest on relative inter-group differences corroborated by blebbistatin-independent histological endpoints, rendering a pharmacological artifact unlikely. Future studies combining genetically encoded Ca^2+^ indicators with computational motion compensation would further circumvent this limitation (Li et al., 2026).

Soattin, D’Souza and colleagues recently demonstrated in a canine model that sustained endurance exercise increases the emergence of triggered activity at the pulmonary veins (Soattin et al., 2026). The delayed afterdepolarizations (DADs) underlying such triggers represent cellular-level arrhythmogenic events that are closely linked to disturbances in intracellular calcium handling. This observation lends independent support to our findings, corroborating both the aberrant expression of calcium-regulatory genes and the abnormalities in calcium handling indices documented in the present study.

## 5 Conclusion

In conclusion, this study offers a comprehensive characterization of exercise dose-dependent atrial remodeling and AF susceptibility in a murine forced treadmill model. High-dose exercise was associated with a heightened imbalance of autonomic modulation and with disturbances in atrial calcium handling, including accelerated calcium release kinetics, impaired diastolic restitution, and enhanced calcium alternans. Together, these findings establish a consistent association linking high-dose exercise to atrial remodeling, dysregulated calcium-related transcriptomic signatures, and increased AF inducibility. Collectively, they position calcium handling instability as a plausible contributor to the arrhythmogenic substrate underlying exercise-related atrial fibrillation.

### 5.1 Study limitations

Several limitations should be acknowledged. First, although the murine model is well suited for mechanistic inquiry, it may not fully recapitulate human atrial electrophysiology; validation in larger animal models and clinical cohorts would strengthen translational relevance. Second, the absence of continuous electrocardiographic monitoring may have limited our ability to capture spontaneous arrhythmias in relation to exercise dose. Third, the present study did not incorporate a systematic sex comparison. Given the known modulatory effects of sex hormones on cardiac Ca^2+^ handling and arrhythmia susceptibility, the generalizability of the observed dose-dependent effects across sexes warrants further investigation. Fourth, optical mapping was performed ex vivo in Langendorff-perfused hearts, thereby excluding neurohormonal and hemodynamic modulation; moreover, blebbistatin precluded assessment of mechanoelectrical feedback, such that our findings pertain to arrhythmogenic substrate and inducibility rather than spontaneous arrhythmia initiation. Fifth, the high-dose group displayed features of systemic stress, including reduced weight gain and diminished food intake, suggesting that the atrial phenotype may reflect combined effects of exercise load and systemic stress; future studies incorporating workload-matched controls and stress biomarker assessment would help disentangle these contributions.

## Data Availability

The datasets presented in this study can be found in online repositories. The names of the repository/repositories and accession number(s) can be found below: https://www.ncbi.nlm.nih.gov/, PRJNA1503657.
