# Supplementary material for: Dose-dependent exercise promotes the transition from atrial protection to arrhythmogenic vulnerability: the intermediary role of calcium handling instability in substrate remodeling
Source: Front Physiol. 2026 Aug 31;17:1878610. doi: 10.3389/fphys.2026.1878610 (PMC13573242; doi:10.3389/fphys.2026.1878610)
Supplement: Supplementary file 1 [file Table1.docx]

Table S1. **Exercise Behavior Scoring Scale.**

| **Behavioral Indicator** | **Scoring Criteria** | **Score** |
| --- | --- | --- |
| **1.Voluntary locomotor activity** |  |  |
| Highly Motivated | Actively runs with speed synchronized to the treadmill, natural posture, no external stimulation required | 4 |
| Motivated | Capable of sustained running, occasionally (<5 times/10 minutes) requires mild stimulation (tail touch) | 3 |
| Moderate | Requires frequent stimulation (5 - 10 times/10 minutes) to maintain running | 2 |
| Unmotivated | Frequent stops (>10 times/10 minutes), requires continuous stimulation | 1 |
| Refusal to Exercise | Completely uncooperative, lies prone on the treadmill or repeatedly contacts the electric shock grid | 0 |
| **2. Exercise Endurance** |  |  |
| Excellent | Maintains stable running throughout with no obvious signs of fatigue | 4 |
| Good | Stable running for the initial 80% of the duration, with mild fatigue appearing in the later stage | 3 |
| Moderate | Stable for the first 50% of the duration, followed by noticeable fatigue and a decrease in speed | 2 |
| Poor | Acceptable for the first 30% of the duration, followed by severe fatigue and frequent rest periods | 1 |
| Very Poor | Shows fatigue shortly after onset, unable to complete the scheduled duration | 0 |
| **3. Motor Coordination** |  |  |
| Excellent | Coordinated gait, smooth limb movement, and good postural balance | 4 |
| Good | Generally coordinated gait, with occasional minor instability | 3 |
| Moderate | Poorly coordinated gait, frequent swaying or leaning | 2 |
| Poor | Disorganized gait, with frequent loss of balance | 1 |
| Very Poor | Unable to maintain a normal gait, frequent falls | 0 |
| **4. Stress Response** |  |  |
| No | Relaxed state, steady breathing, no defecation or urination | 4 |
| Mild | Slightly tense, mildly increased respiratory rate, occasional defecation (1 - 2 times) | 3 |
| Moderate | Clearly tense, rapid breathing, multiple defecations (3 - 5 times) | 2 |
| Severe | Extremely tense, shows signs of respiratory distress, frequent defecation/urination (>5 times) | 1 |
| Critical | State of panic, vocalizations, incontinence, requires immediate termination of the experiment | 0 |
| **5. Respiratory Rate** |  |  |
| Normal | 90 - 220 breaths/min, returns to resting level within <1 minute | 4 |
| Mild Increase | 220 - 260 breaths/min, recovers within 1 - 3 minutes | 3 |
| Moderate Increase | 270 - 310 breaths/min, recovers within 3 - 5 minutes | 2 |
| Severe Increase | 310 - 350 breaths/min, recovers within 5 - 10 minutes | 1 |
| Severe Respiratory Distress | >350 breaths/min or <60 breaths/min, fails to recover within >10 minutes, or exhibits open-mouth breathing | 0 |
| **6. Fur Quality** |  |  |
| Normal Gloss | Smooth, glossy, sleek coat, no significant change from the pre-exercise state | 4 |
| Slight Piloerection | Slight piloerection in localized areas (e.g., neck/back), no moisture | 3 |
| Obvious Piloerection | Widespread piloerection (“puffed up” appearance), slight moisture in some areas | 2 |
| Piloerection with Moisture | Full-body piloerection, visibly moist (from sweat or saliva), loss of gloss | 1 |
| Severely Abnormal | Coat soaked, matted, or shows significant fur loss | 0 |
| **7. Ocular Condition** |  |  |
| Normal | Bright, alert eyes, normal pupil size, no discharge, eyelids fully open | 4 |
| Mild Abnormality | Slightly tired appearance, mild pupil dilation, no discharge | 3 |
| Moderate Abnormality | Dull gaze, obvious pupil dilation or constriction, partially closed eyelids, slight discharge | 2 |
| Severe Abnormality | Dull or tightly closed eyes, abnormal pupils (pinpoint or dilated), significant discharge or tear staining | 1 |
| Critical Abnormality | Eyes tightly shut and cannot be opened, profuse discharge, exophthalmos or sunken eyes, absent pupillary light reflex | 0 |
| **8. Mental State** |  |  |
| Good Spirits | Active and alert, immediately explores the environment, responds briskly to external stimuli, voluntarily drinks and eats | 4 |
| Fair Spirits | Relatively calm, appears slightly tired but still exhibits exploratory behavior, responds to stimuli, drinks water | 3 |
| Moderate Spirits | Reduced activity, tends to curl up or lie still, sluggish response to stimuli, does not voluntarily drink or eat | 2 |
| Depressed | Lethargic, refuses to move, minimal response to stimuli, refuses food and water | 1 |
| Critical | Stuporous or comatose, no response or abnormal response (e.g., aggression/fear) to stimuli, requires emergency intervention | 0 |
